# The genetic architecture of phosphorus efficiency in sorghum involves pleiotropic QTL for root morphology and grain yield under low phosphorus availability in the soil

**DOI:** 10.1186/s12870-019-1689-y

**Published:** 2019-02-28

**Authors:** Karine C. Bernardino, Maria Marta Pastina, Cícero B. Menezes, Sylvia M. de Sousa, Laiane S. Maciel, Geraldo Carvalho Jr, Claudia T. Guimarães, Beatriz A. Barros, Luciano da Costa e Silva, Pedro C. S. Carneiro, Robert E. Schaffert, Leon V. Kochian, Jurandir V. Magalhaes

**Affiliations:** 1 Embrapa Milho e Sorgo, Rodovia MG 424, km 65, Caixa Postal 151, Sete Lagoas, MG 35701-970 Brazil; 2 Universidade Federal de Viçosa, Avenida Peter Henry Rolfs, s/n, Viçosa, MG 36570-900 Brazil; 3 Departamento de Biologia Geral, Universidade Federal de Minas Gerais, Avenida Presidente Antônio Carlos, 6627, Belo Horizonte, MG 31270-901 Brazil; 4 Global Institute for Food Security, University of Saskatchewan, Saskatoon, SK S7N 4J8 Canada; 5 Present Address: Helix Sementes, Rua Arnaldo Luiz de Oliveira, 75, Setor D, Bela Vista, Patos de Minas, MG 38703-240 Brazil

**Keywords:** Phosphorus deficiency, Phosphorus stress, Acid soils, Root system architecture

## Abstract

**Background:**

Phosphorus (P) fixation on aluminum (Al) and iron (Fe) oxides in soil clays restricts P availability for crops cultivated on highly weathered tropical soils, which are common in developing countries. Hence, P deficiency becomes a major obstacle for global food security. We used multi-trait quantitative trait loci (QTL) mapping to study the genetic architecture of P efficiency and to explore the importance of root traits on sorghum grain yield on a tropical low-P soil.

**Results:**

P acquisition efficiency was the most important component of P efficiency, and both traits were highly correlated with grain yield under low P availability. Root surface area was positively associated with grain yield. The guinea parent, SC283, contributed 58% of all favorable alleles detected by single-trait mapping. Multi-trait mapping detected 14 grain yield and/or root morphology QTLs. Tightly linked or pleiotropic QTL underlying the surface area of fine roots (1–2 mm in diameter) and grain yield were detected at positions 1–7 megabase pairs (Mb) and 71 Mb on chromosome 3, respectively, and a root diameter/grain yield QTL was detected at 7 Mb on chromosome 7. All these QTLs were near sorghum homologs of the rice serine/threonine kinase, *OsPSTOL1*. The *SbPSTOL1* genes on chromosome 3, *Sb03g006765* at 7 Mb and *Sb03g031690* at 60 Mb were more highly expressed in SC283, which donated the favorable alleles at all QTLs found nearby *SbPSTOL1* genes. The Al tolerance gene, *SbMATE*, may also influence a grain yield QTL on chromosome 3. Another *PSTOL1*-like gene*, Sb07g02840*, appears to enhance grain yield via small increases in root diameter. Co-localization analyses suggested a role for other genes, such as a sorghum homolog of the *Arabidopsis ubiquitin-conjugating E2 enzyme*, *phosphate 2* (*PHO2*), on grain yield advantage conferred by the elite parent, BR007 allele.

**Conclusions:**

Genetic determinants conferring higher root surface area and slight increases in fine root diameter may favor P uptake, thereby enhancing grain yield under low-P availability in the soil. Molecular markers for *SbPSTOL1* genes and for QTL increasing grain yield by non-root morphology-based mechanisms hold promise in breeding strategies aimed at developing sorghum cultivars adapted to low-P soils.

**Electronic supplementary material:**

The online version of this article (10.1186/s12870-019-1689-y) contains supplementary material, which is available to authorized users.

## Background

Sorghum is a versatile crop that was domesticated in the tropics, in the northeastern quadrant of the African continent, possibly at least 5000 years ago [1]. Along with pearl millet, sorghum is the main staple food crop of the West African Savannah zones and in that region, guinea sorghums are broadly adapted to different stresses, including those caused by poor soil fertility [2]. In sub-Saharan Africa, two of the most important abiotic stresses that limit sorghum production are Al toxicity and low-P availability in the soil [3–5].

Both types of abiotic stresses share a common chemical basis centered on the prevalence of Al and Fe oxides in the clay fraction of highly weathered tropical soils [6]. Under low pH, Al is hydrolyzed into the ionic form, Al^3+^, which damages plant roots, reducing crop yields [7]. Low-P availability, in turn, results from P fixation with Al and Fe oxides [8]. Plant roots absorb P from the soil solution in the orthophosphate forms, H_2_PO_4_^−^ and HPO_4_^2−^ [9]. However, P fixation into soil clays impairs P diffusion from the soil solution towards the root surface, restricting uptake. Approximately half of the world agricultural lands have low-P availability [10]. Even in high input production systems, the non-renewable nature of phosphatic rock fertilizer [11] raises questions regarding the sustainability of continuously increasing rates of P fertilizer applications, which are needed to sustain crop yields. Therefore, in view of the prevalence of low-P soils in agricultural frontiers in which food production needs continuous improvement, such as in Africa, the identification of genetic factors that can be used to facilitate breeding for sorghum adaptation to low-P conditions become of utmost importance for global food security.

Aluminum tolerance in sorghum is due to the action of the Al-induced and Al-activated root citrate transporter, *SbMATE*, which underlies the aluminum tolerance locus, *Alt*_*SB*_, at the terminal region of sorghum chromosome 3 [12]. Recently, the *SbMATE* allele donated by the guinea sorghum, SC283, has been shown to enhance sorghum grain yield by over 1.0 ton ha^− 1^ on an acid, Al toxic soil, with no detectable yield penalty in the absence of Al toxicity [13]. Leiser et al. [5], using *Alt*_*SB*_-specific markers, also found strong associations of *SbMATE* with grain yield production, particularly in low-P conditions in many environments in West-Africa. This suggests that *SbMATE* confers P use efficiency (PUE) in addition to Al tolerance, possibly via a joint effect of citrate mobilizing P that is fixed on the soil clays [14], and by enhancing root development in Al tolerant genotypes, increasing P uptake [15].

The ability of a plant to grow and to produce reasonable levels of grain and biomass under low-P availability, which we designate here as P use efficiency (PUE, or simply P efficiency), can be achieved via different mechanisms acting to optimize utilization of internal P or to enhance P acquisition [16]. From the crop physiology standpoint, these mechanisms may result from the modulation of P transporters, organic acid exudation, phosphatase secretion, mycorrhizae associations and alterations in root system architecture in response to low-P conditions, among other mechanisms (reviewed by López-Arredondo et al. [17]). For maize cultivated on a tropical low-P soil, P acquisition has been reported to be more important than P internal utilization to explain differences in P use efficiency [16], which was also confirmed by QTL mapping results [18]. These studies emphasize the importance of changes in root system architecture and morphology as a mechanism favoring P acquisition (reviewed by Magalhaes et al. [19]). These modifications may involve changes in lateral root growth and angle, presence of shallow roots, in addition to enhanced proliferation of root hairs [10, 17, 20].

There is a recent body of evidence suggesting that genes modulating root morphology may result in increased P efficiency. Overexpression of the rice serine/threonine receptor-like kinase, *phosphorus starvation tolerance1* (*OsPSTOL1*, [21]) has been shown to increase grain yield in rice cultivated on a low-P soil via *OsPSTOL1*-elicited enhancement of early root growth, which favors P uptake in the developing rice plant. Subsequently, association mapping established that allelic variation at homologs of *OsPSTOL1* in sorghum, designated as *SbPSTOL1* genes, was associated with enhanced grain yield production on a low-P soil, likely via changes in root morphology, particularly root diameter and root surface area [22]. In addition, recent studies in Arabidopsis suggested a role in P efficiency for genes involved with Al tolerance, such as the malate transporter*, ALMT1* [23] and its regulatory factor, the C_2_H_2_-type zinc finger, sensitive to proton rhizotoxicity 1 (AtSTOP1 [24]), in addition to the ABC-like transporter, aluminum sensitive 3 (*ALS3* [25, 26]). These genes appear to mediate an iron-dependent mechanism leading to enhancement of lateral root growth [27–31], which can possibly increase P uptake on acidic soils [15].

Using a genetic approach based on multi-trait QTL mapping, the present study aimed at unravelling the genetic architecture of P efficiency in a large sorghum recombinant inbred line population, and to establish links between the genetics and physiology of P efficiency, such as associations between root morphology, P content and sorghum grain yield on soils with low-P availability.

## Results

### Phenotypic analyses in the parents and RIL population

The most important trait for P efficiency within a breeding context, grain yield in the field, was assessed under low-P availability in the soil. We also estimated the relative contributions of the efficiency at which a plant acquires P from the soil (P acquisition efficiency, PAE) and also internal utilization efficiency (PUTIL), on overall P use efficiency (PUE or simply P efficiency, that encompasses both PAE and PUTIL) [16, 32]. Table 1 shows that PAE was the most important component influencing PUE for sorghum cultivated under low-P availability in the soil. Acquisition efficiency accounted for 82% of the variability in PUE, whereas the contribution of the PUTIL component was comparatively much smaller (18%). Therefore, we also assessed root morphology in hydroponics as changes in root morphology including increased root length can lead to enhanced P uptake and grain yield in soils with low-P availability. To gain insights into sorghum performance in hydroponics, we also assessed dry matter accumulation (DM) and shoot and root P content.

**Table 1:** Relative importance of PAE and PUTIL over PUE assessed in low-P conditions field

Trait	Correlation *(r*_*xiy*_ *)* ^*a*^	Standard Deviation (S)	*Sx*_*i*_ */S*_*y*_	Relative importance
*PAE (x1)*	0.9216	0.2285	0.8868	0.82
*PUTIL (x2)*	0.4763	0.0999	0.3878	0.18
*PUE (y)*		0.255		

^a^*rxiy*: Phenotypic correlation among Phosphorus acquisition efficiency (PAE) and Phosphorus internal utilization efficiency (PUTIL) and Phosphorus use efficiency (PUE)

We observed substantial genetic variance for all traits assessed in the present study, with heritability estimates ranging from 0.3 (root diameter - RD) to 0.8 (plant height - PH, Additional file 1). Traits reflecting sorghum performance grown on low-P growth media measured in nutrient solution (DM and P content) and in the field (grain yield - Gy) showed intermediate to high heritability estimates of between 0.4 and ~ 0.8, indicating reasonable experimental precision to detect regions of the sorghum genome associated with P efficiency. Marked transgressive segregation for grain yield in the recombinant inbreed line (RIL) population, where a maximum of 4.5 ton ha^− 1^ exceeded by more than two-fold the grain yield for either parent (Additional file 1), emphasizes the rather complex, polygenic nature of P efficiency measured in sorghum cultivated under low-P availability in the soil.

**Fig. 1:**
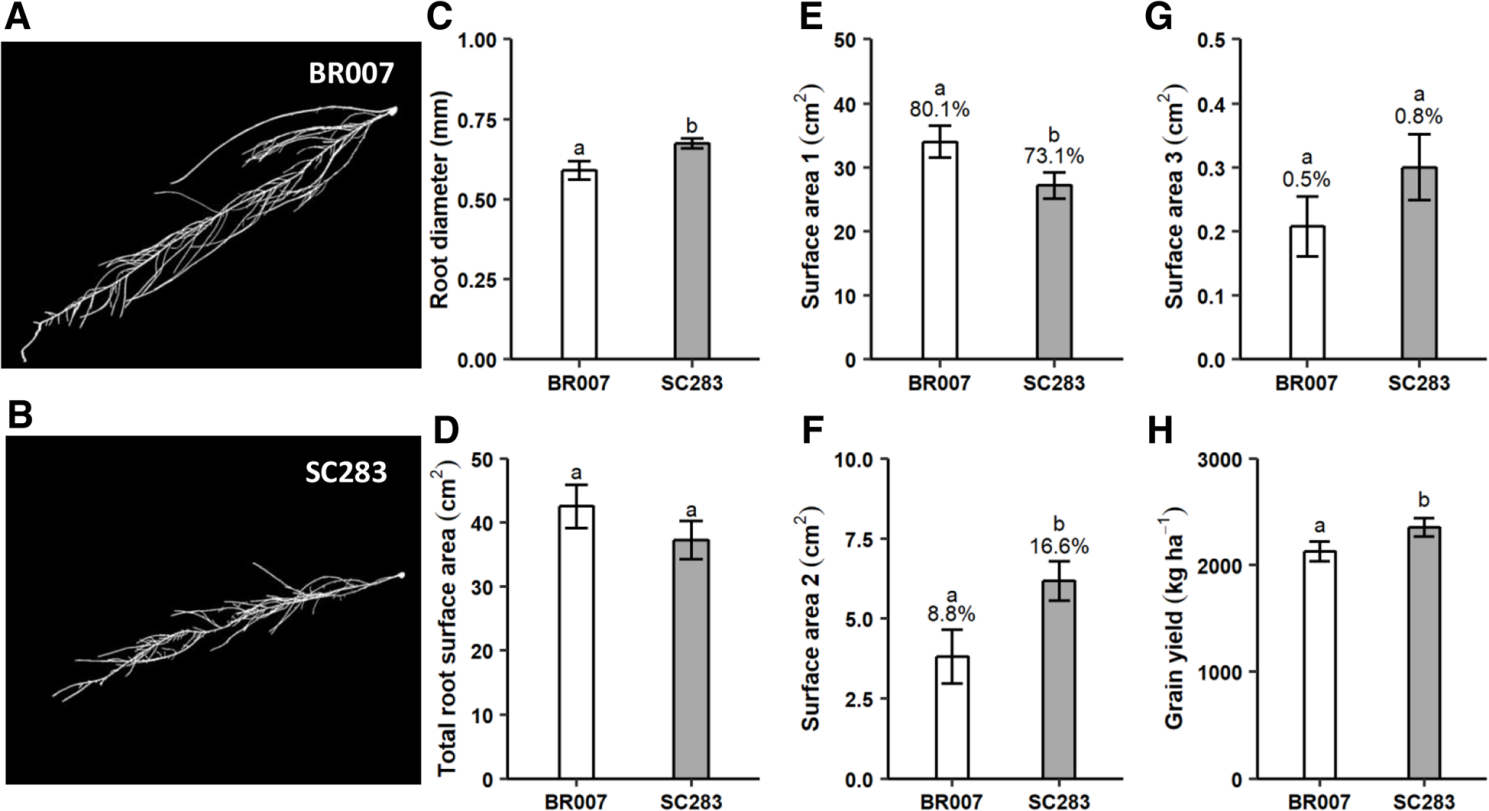
Phenotypic characterization of the RIL parents. Images of the (**a**) BR007 and (**b**) SC283 root systems. Phenotypic means for **(c)** root diameter (RD), **d** total root surface area (SA), surface area of roots in the diameter classes of **(e)** 0–1 mm, (SA1, designated as very fine roots), **f** 1–2 mm (SA2, fine roots) and **(g)** 2–4.5 mm (SA3, thicker roots). All surface area measures are in cm^2^. Root images and root morphology traits were assessed after 13 days in nutrient solution with low-P. **h** Grain yield (Gy) was assessed in a low-P soil with one hundred twenty reps. The proportion of roots within each diameter class relative to total surface area of the root systems are shown as percentages in **e**, **f** and **g.** Error bars are shown. Different letters indicated statistical differences by the t-test (*p*-values ≤ 0.10)

We measured total root surface area (SA) of the sorghum root system and also the root surface area of roots within the diameter classes of 0–1, 1–2 and 2–4.5 mm, which are designated hereafter as very fine, fine and thicker roots, respectively. BR007 tended to exhibit greater total root surface area and had thinner roots compared to SC283 (Fig. 1a-d), which is due to the prevalence in BR007 of roots in the 0–1 mm diameter class (Fig. 1e - labeled SA1). These very fine roots comprise most of the root system in both parents but are more prevalent in BR007 (80%) compared to SC283 (73%) (Fig. 1e). However, when measured in the different root diameter classes, root surface area turned out to be heterogeneous between the parents, with SC283 showing higher surface area of both fine (SA2) and thicker (SA3) roots (Fig. 1f-g) compared to BR007. However, fine roots are still far more prevalent (~ 17%) than thicker roots (~ 1%) in the SC283 root system. Finally, the most important trait to reflect P efficiency, grain yield under low-P availability in the soil, was approximately 12% higher for the guinea race parent, SC283, compared to BR007 (Additional file 1 and Fig. 1h).

### Trait associations

PAE and PUE were both highly correlated with grain yield (r = 0.85 and 0.97, respectively; Additional file 2), which is consistent with the importance of P acquisition on P use efficiency (Table 1). PUTIL, which we found to be a minor component of PUE compared to acquisition efficiency (Table 1), was less correlated with grain yield (*r* = 0.4).

**Fig. 2:**
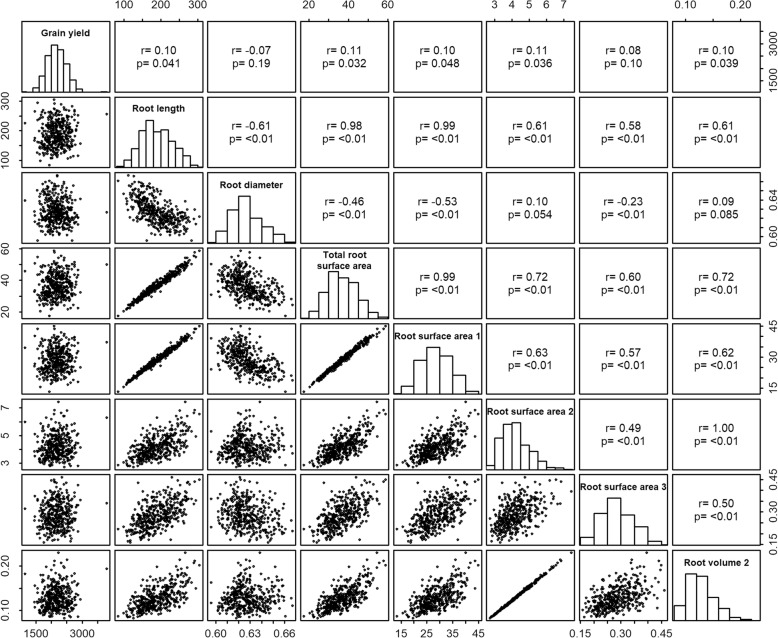
Genetic correlation analysis of grain yield and root morphology traits. Grain yield data (kg ha^− 1^) was acquired for plants grown on a low-P soil. The root morphology traits assessed after 13 days in nutrient solution with low-P are total root length (cm), root diameter (RD), total root surface area (SA), surface area of roots in the diameter classes of 0–1 mm (SA1, designated as very fine roots), 1–2 mm (SA2, fine roots) and 2–4.5 mm (SA3, thicker roots), and volume of fine roots between 1 and 2 mm in diameter (root volume 2, in cm^3^). Surface area measurements are expressed in cm^2^. Pearson correlation coefficients (r) and *p*-values (p) are shown

Next, we studied the association between root morphology traits and grain yield under low-P availability in the soil via a genetic correlation analysis (Fig. 2). Total root surface area was highly correlated with total root length (correlation coefficient, r = 0.98) and surface area of very fine roots (SA1) (*r* = 0.99). In addition, surface area of fine roots (SA2) was highly correlated with root volume 2 (*r* = 1.0). Therefore, among those traits, root surface area was used to gain insights into the role of root morphology on grain yield under low-P availability in the soil. A reduction in root diameter was in general associated with increased total root surface area (*r* = − 0.46), which was driven primarily by very fine roots (RD vs. SA1, *r* = − 0.53) and, to a lesser extent, by thicker roots (RD vs. SA3, *r* = − 0.23). This suggests the existence of some genetic determinants that act to increase root surface area via enhanced development of finer roots. However, the magnitude of the correlation coefficients also indicates that root surface area and root diameter are controlled to some extent independently. Surface area of fine roots was positively but weakly correlated with root diameter (RD vs. SA2, *r* = 0.1), suggesting that slight increases in root diameter between 1 and 2 mm may result in enhanced surface area. Grain yield under low-P availability in the soil was significantly correlated with the different traits reflecting surface area of fine roots (*r* ≅ 0.1, *p*-value < 0.05), although this association tended to dissipate with thicker roots between 2 and 4.5 mm in diameter (SA3, *r* = 0.08, *p*-value = 0.10).

### QTL mapping for root morphology and performance traits under low-P

We mapped QTLs underlying P efficiency traits and found that the majority of the QTLs, primarily for PAE (9 out of 10) and PUE (9 out of 10), but also for PUTIL (although to a lesser extent) coincided with those detected for grain yield, with exception of the QTL on chromosome 5 for PUTIL (Additional file 3). This is consistent both with the much higher importance of PAE compared to PUTIL on PUE (Table 1) and with the strong association between grain yield and PAE/PUE (Additional file 2).

Although grain yield was the most informative trait for QTL detection, a PUTIL QTL on chromosome 5 may harbor genes underlying changes in P internal utilization and two chromosome 1 QTLs may jointly underlie PAE and PUTIL. As PUTIL was much less important than P acquisition efficiency for PUE, we thus focused primarily on the genetic mechanisms that enhance P acquisition efficiency via changes in root morphology and their role in increased grain yield on low-P soil.

**Fig. 3:**
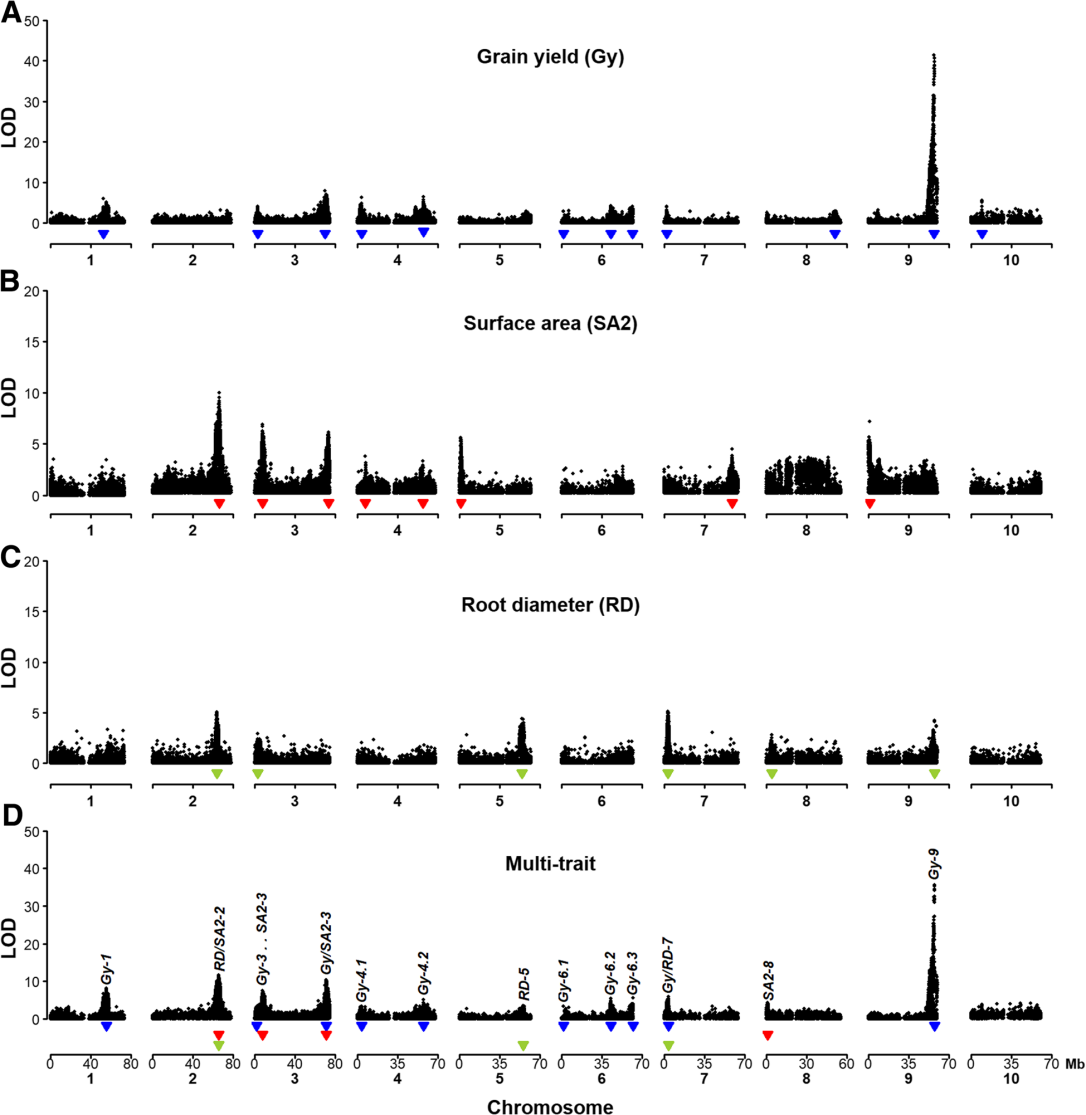
Single- and multi-trait QTL mapping profiles for grain yield and root morphology traits. The final set of traits used for multi-trait QTL mapping was comprised of grain yield (Gy), surface area of fine roots in the 1–2 mm diameter class (SA2) and root diameter (RD). Grain yield (Gy) data (kg ha^− 1^) was acquired in a low-P soil. The root morphology traits assessed after 13 days in nutrient solution with low-P are root diameter (RD, mm), surface area of fine roots between 1 and 2 mm in diameter (SA2, in cm^2^). QTL profiles obtained with (**a-c**) single- and (**d**) multi-trait QTL mapping are shown. QTLs were designated based on the respective traits followed by the chromosome locations, and are numbered in the case of multiple QTLs within the same chromosome. For example *Gy-6.1* is a grain yield QTL located in the beginning of chromosome 6. Tight linkage between QTL or possible pleiotropy were depicted by double dots and slashes, respectively in the QTL designations. Blue, red and green inverted triangles depict the positions of QTLs for Gy, SA2 and RD, respectively

We initially conducted single-trait QTL mapping with many different traits related to root morphology and sorghum performance under low-P conditions (Additional file 4). This analysis detected a total of 101 QTLs, with the favorable allele of 59 QTLs donated by the guinea parent, SC283, whereas the BR007 alleles increased phenotypic expression for 42 QTLs. Based on the correlation analyses between traits and on the single-trait QTL mapping results, we selected a subset of non-redundant and highly informative traits (i.e. traits repeatedly associated with some QTLs) for multi-trait QTL mapping, focusing primarily on the most important P efficiency trait, namely grain yield under low-P availability in the soil (Fig. 3). P content in the grain (Pg), for example, was highly correlated with grain yield (*r* = 0.92) and we thus we only included grain yield and not Pg for multi-trait QTL mapping. The final set of traits used for multi-trait QTL mapping was comprised of grain yield (Gy), surface area of fine roots in the 1–2 mm diameter class (SA2) and root diameter (RD).

For the selected traits, the majority of the QTLs detected by single-trait QTL mapping (Fig. 3a-c) were also detected by multi-trait QTL mapping (Fig. 3d). Exceptions are the QTLs for SA2 on chromosomes 5, 7 and 9 and the Gy QTL on chromosome 8 and 10, which were not detected using multi-trait QTL mapping. Multi-trait mapping detected 14 QTLs (see Fig. 3a-c for single-trait mapping results) and revealed ten QTLs related to grain yield (Fig. 3d), within which one QTL was tightly linked to a root morphology QTL (*Gy-3*…*SA2–3*) and two were possibly pleiotropic with root morphology (*Gy/SA2–3* and *Gy/RD-7*). For all of these QTLs, the favorable allele was donated by SC283 (Additional file 5). In contrast, the favorable alleles for five of the eight grain yield-specific QTLs were donated by BR007.

The different grain yield QTLs explained, in general, approximately 1 to 5% of the genetic variance and increased grain yield by ~ 120 kg ha^− 1^ (Additional files 4 and 5), except for a Gy QTL at the end region of chromosome 9 (*Gy-9*). This QTL was detected for several different traits (Additional file 4), explained the largest proportion of the genetic variance (~ 26%, Additional file 4), and was associated with the largest increase in grain yield, of ~ 400 kg ha^− 1^, with the favorable allele donated by BR007.

**Fig. 4:**
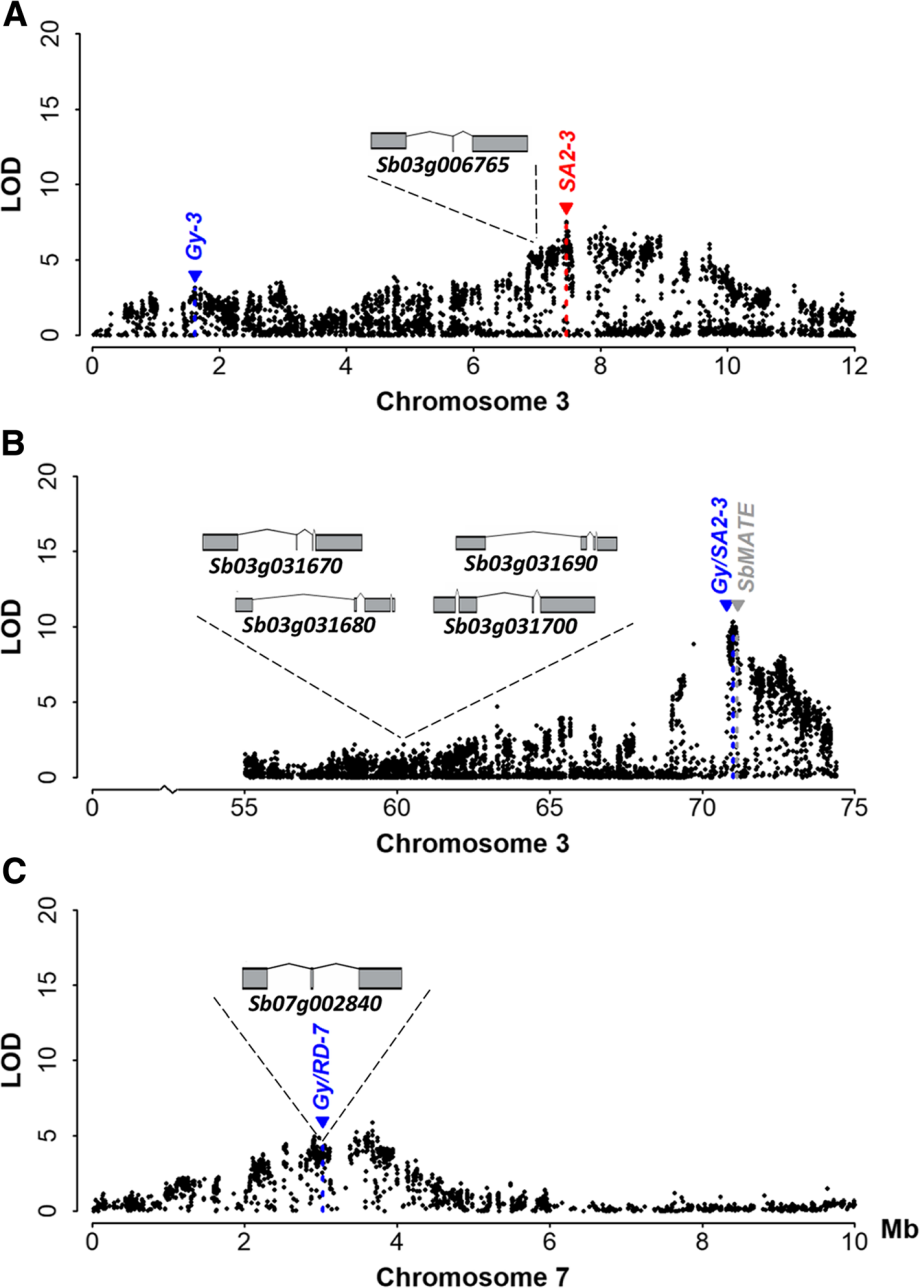
Physical positions of *SbPSTOL1* genes in the context of the QTL regions detected by multi-trait QTL mapping (Fig. 3d). The root morphology traits assessed after 13 days in nutrient solution with low-P are root diameter (RD, mm) and surface area of fine roots between 1 and 2 mm in diameter (SA2, in cm^2^). Possible pleiotropy between Gy (grain yield), SA2 and RD QTL, when detected, were depicted by slashes in the QTL designations. Physical positions and gene models for the *SbPSTOL1* genes (https://phytozome.jgi.doe.gov/, v1.4 of the sorghum genome), **a** *Sb03g006765*, **b** the *SbPSTOL1* cluster including *Sb03g031690* and (**c**) *Sb07g002840* are shown

Based on single-trait QTL analysis, all grain yield QTLs detected by multi-trait QTL mapping were co-located or were found near QTLs underlying P content and/or dry matter accumulation in hydroponics under low-P (Fig. 3, Additional file 4). The *RD/SA2–2* QTL (Additional file 5), which was the only root morphology QTL not associated with grain yield, co-located with QTLs for root dry matter accumulation, and shoot and root P content assessed in hydroponics via single-trait analyses (Additional file 4). Multi-trait QTL mapping provided insights into possible pleiotropic QTLs underlying changes in root system morphology and grain yield in the context of genes previously shown to be associated with those traits, such as sorghum homologs of the rice serine/threonine kinase, *OsPSTOL1* [22]. The physical positions of the *SbPSTOL1* genes and that of *SbMATE*, which confers sorghum Al tolerance [33], in the context of the QTL detected by multi-trait QTL mapping, are shown in Fig. 4. The QTLs *Gy-3* and *SA2–3* were in close physical proximity, between 5.38 and 0.46 Mb, respectively, from the *PSTOL1* gene *Sb03g006765* (Fig. 4a). At the end of chromosome 3, a cluster of four *SbPSTOL1* genes were located ~ 11 Mb from the *Gy/SA2–3* QTL and this QTL was only 80 Kb from *SbMATE* (Fig. 4b). Finally, the *Gy/RD-7* QTL is located only 0.66 Mb from the *SbPSTOL1* gene, *Sb07g002840* (Fig. 4c).

### Expression profile of *SbPSTOL1* genes in the parent’s root systems under low-P

**Fig. 5:**
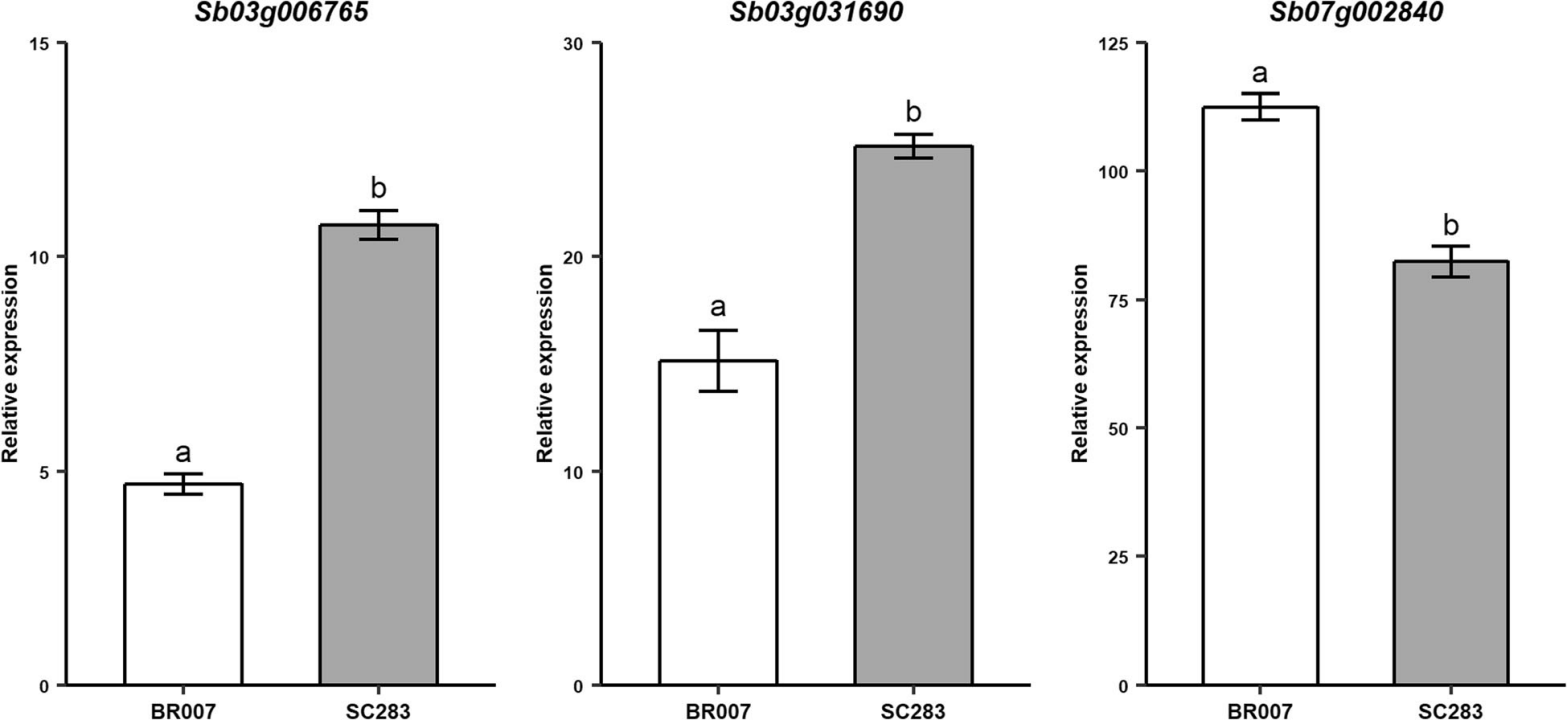
Expression profile of *SbPSTOL1* genes found near QTLs for grain yield and root morphology (Fig. 3d) via quantitative RT-PCR. Whole root systems of the parents cultivated in nutrient solution with low-P for 13 days were collected, frozen, and used for quantitative RT-PCR. Expression was assessed using the 2^-ΔΔCT^ method [69]. Different letters indicated statistical differences by the t-test (*p*-values ≤0.05). Error bars are shown

Multi-trait QTL mapping results indicated that the favorable alleles at QTLs either tightly linked or possibly pleiotropic with grain yield and root morphology on chromosomes 3 and 7, which were located in the vicinity of *SbPSTOL1* genes, were consistently donated by SC283. Next, we assessed the expression profile of these *SbPSTOL1* genes in roots of the RIL parents, BR007 and SC283, subjected to low-P conditions in hydroponics. *Sb03g006765*, located at the beginning of chromosome 3, and *Sb03g031690*, which is part of a *SbPSTOL1* cluster at position ~ 60 Mb on chromosome 3 [22], were both more highly expressed in the roots of the guinea parent, SC283, in the low-P growth media (Fig. 5). In contrast, expression of *Sb07g002840* was higher in BR007 roots, which donates the inferior allele at the *Gy/RD-7* QTL.

## Discussion

Low-P availability in the soil is a major factor that compromises food security in many developing countries in West Africa that rely on the sorghum crop for food production [2]. West Africa is the primary domestication center of the guinea race of sorghum [34], which is used therein as a pivotal staple food in areas with low soil fertility [35]. Thus, sorghum adaptation to soils with low-P availability becomes critical for food security [5, 35].

Our QTL mapping study emphasized the complex nature of traits related to P efficiency in sorghum, with favorable alleles donated by both parents in rather equal proportions. However, the observed slight overrepresentation of superior QTL alleles derived from the guinea race parent, SC283, may not be coincidental, reflecting local adaptation of guinea sorghums to poor soil fertility and acid soils in West Africa [2].

### QTLs for root morphology coincide with grain yield QTL under low-P availability

The root system of monocotyledonous crop plants consists of one or more seminal roots that originate from the seed embryo after germination, and crown roots that emerge later from nodes along the stem [36]. Increased root surface area, which can be achieved via enhanced lateral root branching, can enhance P uptake and plant growth [37]. Among the ten grain yield QTL that were detected by multi-trait mapping, three were either tightly linked (one QTL) or possibly pleiotropic (two QTL) with root morphology traits. Those are: 1) the grain yield QTL, *Gy-3* and the QTL for surface area of fine roots, *SA2–3* at the beginning of chromosome 3, which are only ~ 6 Mb apart; 2) the pleiotropic *Gy/SA2–3* QTL at position ~ 71 Mb on chromosome 3; and 3) the *Gy/RD-7* QTL at 3.6 Mb on chromosome 7 (Fig. 3d). Thus, our multi-trait QTL mapping approach established an important role for root system morphology as an entry point for molecular breeding strategies targeting enhanced P uptake and grain yield under low-P availability in the soil.

### Specific changes in root morphology are likely important for P efficiency

The grain yield QTLs that are possibly determined by changes in root surface area seem to be more specific to roots between 1 and 2 mm (SA2) in diameter than to the very fine roots between 0 and 1 mm (SA1) or thicker roots (2–4.5 mm, SA3). Via single-trait mapping, a QTL for SA2 (and not for other root diameter classes) was found at the beginning of chromosome 3, tightly linked to a Gy QTL, whereas the grain yield/surface area QTL at the end of chromosome 3 was near SA2 and SA3 QTL. A total surface area (SA) QTL was also found at the end of chromosome 3, but this is expected as total surface area, which is highly correlated with SA1, largely represents the sum of surface area of roots in all diameter classes. Importantly, eight QTLs in total were detected for surface area of fine roots via single-trait mapping, whereas only four and two QTLs were detected for total surface area (that is highly correlated with surface area of very fine roots, r = 0.99) and for surface area of thicker roots, respectively. Very thick roots are not expected to play a major role in nutrient uptake, as plant species with a majority of fine roots in their root systems tend to optimize the ratio between root surface area available for uptake and root weight, reflecting a reduced carbon cost for root biomass formation [38].

It is generally thought that the finer the roots, the better the root system can mine the soil for diffusion-limited nutrients, like the phosphate anion on tropical soils. However, although fine roots are the key factor for uptake, particularly for nutrients with very low mobility in tropical soils such as P, our QTL data interestingly suggest there is a trade-off between decreased root diameter and enhanced P uptake. This can be expected as decreased root diameter, beyond a given threshold may, for example, limit root penetration through the soil [38] and, possibly, lead to less root longevity [39].

### *SbPSTOL1* genes possibly underlie QTLs for root morphology and grain yield

All three root morphology QTL that were found to be either tightly linked or pleiotropic with grain yield are located in the vicinity of sorghum homologs of the rice serine/threonine kinase, *OsPSTOL1*, which was previously found to enhance early root growth and grain yield in rice under low-P availability [21].

Based on multi-trait mapping, two QTLs underlying grain yield and root surface area, *Gy-3* and *SA2–3*, were found on chromosome 3 at positions 1.6 and 7.4 Mb, respectively. Those QTLs are only ~ 6 Mb apart and are physically very close to the *SbPSTOL1* gene, *Sb03g006765*, at position 7 Mb. A possible pleiotropic QTL, simultaneously underlying SA2 and Gy (*Gy/SA2–3*), was found approximately ~ 11 Mb from a *SbPSTOL1* cluster at position ~ 60 Mb on chromosome 3. The favorable alleles both at the SA2 and Gy QTLs near *Sb03g006765* and at the possible *Gy/SA2–3* pleiotropic QTL at position 71 Mb are derived from the guinea parent, SC283. Although BR007 tended to show greater root surface area compared to SC283, this is due to the prevalence in BR007 of very fine roots, between 0 and 1 mm in diameter (Fig. 1). Compared to BR007, SC283, which donates the positive alleles for the Gy and SA2 QTLs in the vicinity of *SbPSTOL1* genes, has about twice the proportion of fine roots between 1 and 2 mm in diameter, whose surface area gives rise to both SA2 QTL on chromosome 3. Finally, both *Sb03g006765* and *Sb03g031690* (that is part of a *SbPSTOL1* cluster at position ~ 60 Mb), exhibited significantly higher expression in response to low-P growth conditions specifically in SC283 roots, when compared to BR007, which is in agreement with SC283 donating *SbPSTOL1* alleles that enhance the surface area of fine roots at the respective chromosome 3 QTL.

Previously, single nucleotide polymorphism (SNP) loci within the *SbPSTOL1* gene *Sb03g006765*, and in the *SbPSTOL1* genes present in the gene cluster at position ~ 60 Mb, were associated both with variation in root surface area and sorghum performance under low-P [22]. This suggests that the grain yield QTL on chromosome 3 results, at least in part, from enhanced surface area conferred by *SbPSTOL1* genes. However, the major Al tolerance gene, *SbMATE*, is located at position 71 Mb on the same chromosome, and thus is closer to the pleiotropic *Gy/SA2–3* QTL in the region (Fig. 4). *SbMATE* has been shown to contribute to grain yield under low-P conditions, possibly via citrate-based enhanced mobilization of P that is bound to the soil clays [5], or simply as an indirect effect of enhanced root development under Al toxicity in the subsoil [15]. Thus, we cannot rule out that *SbMATE* is responsible for some of the yield advantage that gives rise to the grain yield QTL at the end of sorghum chromosome 3.

We previously reported that allelic variation at the *SbPSTOL1* gene, *Sb07g002840*, influences root diameter, biomass accumulation and P uptake, although associations with grain yield were not found using a sorghum association panel [22]. A pleiotropic QTL underlying both Gy and RD was found only ~ 0.6 Mb away from *Sb07g002840*, and the favorable allele for this *Gy/RD-7* QTL was donated by SC283. It is interesting to note that SC283 exhibits overall a larger root diameter compared to BR007, which is in agreement with the SC283 origin of the favorable allele at the *Gy/RD-7* QTL. In barley subjected to low-P conditions, based on the effects of co-localized QTLs, larger root diameter was related to higher grain yield [40], which is consistent with our results with *Sb07g002840*. A positive relationship has been reported between the size of the apical meristem (reflected by the apical diameter) and important characteristics such as the elongation rate, growth duration and gravitropism [38], which could lead to enhanced performance under low-P conditions. It is thus possible that the slight increases in root diameter elicited by *Sb07g002840* lead to an increase in the surface area of laterals that are still fine, generating more physically robust roots without substantial carbon cost. Hence, these roots would be more efficient to optimize P mining in the soil, leading to enhanced P uptake and grain yield under soil low-P availability. Unlike the *SbPSTOL1* genes on chromosome 3, root *Sb07g002840* expression is lower in SC283 compared to BR007. This could suggest an allele-specific repressor effect of *Sb07g002840* on root diameter, with lower expression of the SC283 allele of *Sb07g002840*, leading to a slight increase in root diameter.

### Synteny analysis in sorghum and maize supports a role for *PSTOL1* genes in root morphology and reveals other genes possibly involved in P efficiency

We compared the positions of the grain yield QTL detected in sorghum by multi-trait QTL mapping with QTLs related to root morphology and P efficiency reported in the closely related species, maize. The summary shown in Additional file 6 was based primarily on a QTL mapping study that included the same traits used in our sorghum RIL population [41] and in a comprehensive meta-analysis of QTLs related to low-P tolerance in maize, which defined 23 consensus QTL (*cQTL*, [42]).

The maize root morphology QTLs, *qMulti3.04*, *qRL8.05* and *qRD4.05*, harbor maize homologs of *Sb03g006765*, *Sb03g031690*, and *Sb07g002840*, respectively (the *SbPSTOL1* genes at chromosomes 3 and 7 that are near grain and root morphology QTLs, Fig. 4), in regions that are syntenic between maize and sorghum (Additional file 6). Consistent with our findings that *Sb07g002840* influences root diameter, *qRD4.05* is also associated with changes in root diameter in maize. Four homologs of *OsPSTOL1* were found in maize *cQTL3–1* at bin 3.04 [42], which overlaps with *qMulti3.04* [41] that is associated with multiple root morphology traits. In this region, the maize *PSTOL1*-like gene, *GRMZM2G412760*, is closely related to *Sb03g006765*. In conjunction with the presence of a functional PSTOL1 protein in rice (*OsPSTOL1*, [21]), this suggests that the PSTOL1 function in modulating root morphology is rather ancient, predating the divergence between maize, sorghum and rice.

This analysis also suggests possible functions for grain yield QTL that are apparently unrelated to root morphology in sorghum. Previously, we detected a major QTL for plant height and flowering time at the end region of sorghum chromosome 9 [43], which coincides with the QTL underlying multiple traits that was found in the present study. The ubiquitin-conjugating enzyme UBC24, encoded by *phosphate2* (*PHO2*), which is a major player in P homeostasis and plant responses to P deficiency [44], has been recently implicated in tolerance to low-P in maize [45]. We found a highly similar *PHO2* homolog at position ~ 57 Mb on sorghum chromosome 9, which closely overlaps with multiple QTLs related to root morphology and many other traits, including grain yield (Additional file 4 and Fig. 3). *GRMZM2G381709*, a maize homolog of *PHO2*, is also located in a syntenic position of the maize genome, near a cQTL (*cQTL6–2*) for low-P tolerance in maize defined by meta-analysis (Additional file 6).

Sorghum homologs of the auxin transporters, PIN1 and PIN6, are found near the grain yield QTL at position ~ 58 Mb on sorghum chromosome 4 (Fig. 3). A related PIN protein in maize is found near another QTL for tolerance to low-P conditions in maize, *cQTL5–5* (Additional file 6). *PIN* genes that encode auxin transporters have been implicated in root architecture changes involving lateral roots under low-P in wheat [46] and may play a significant role in sorghum and maize P efficiency. On chromosome 6, a sorghum homolog of the Al tolerance gene, *ALMT1*, which encodes a root malate efflux transporter and has been recently reported to modulate root growth in response to low-P [30], is found at position ~ 44 Mb, thus near the grain yield QTL at position 42.5 Mb (Fig. 3 and Additional file 4). In maize, the consensus QTL, *cQTL10–1* [42], is also found near a maize homolog of *ALMT1*, suggesting a role for *ALMT1* on P efficiency in both maize and sorghum. As with SbMATE, it is also possible that root malate release via ALMT1 is involved in solubilizing P that is fixed on the surface of Fe and Al oxide minerals in the soil, making them bioavailable for root uptake.

## Conclusions

Phosphorus acquisition efficiency was the major component of P efficiency for sorghum cultivated under low-P availability in the soil and grain yield was highly correlated with both traits. Although our findings emphasize root system morphology as a major target for molecular approaches aimed at developing P efficient sorghum cultivars, other distinct mechanisms may also play a significant role in sorghum performance on low-P soils via enhanced P acquisition. The molecular determinants of such mechanisms, along with *SbPSTOL1* genes, should power novel, gene-based molecular breeding strategies to enhance food security in tropical regions with low-P availability.

## Methods

### Genetic material

A population composed of 396 recombinant inbred lines (RILs, F_10:11_), derived from a cross between the sorghum lines, BR007 and SC283, was developed by single-seed descent [47] at Embrapa Maize and Sorghum (Sete Lagoas - MG, Brazil). Both BR007 (Redbine-type) and SC283 (sorghum converted guinea) were introduced into the Embrapa breeding program in 1972 from the Purdue Breeding Program (West Lafayette - IN, US). BR007 is Al sensitive whereas SC283 is highly tolerant to Al toxicity [33]. Previous studies indicated that, while SC283 has higher grain yield in a soil with low-P availability compared to BR007, the grain yield increase in BR007 in response to adequate P supply is in turn higher than in SC283 [48].

### Phenotyping for low-P in field conditions

Four field experiments were conducted at the experimental station of Embrapa Maize and Sorghum in Sete Lagoas, State of Minas Gerais, Brazil, during the summer season of 2012–2013. The experimental site is a clay and highly weathered tropical soil, with low fertility in natural conditions, low pH, Al toxicity and low-P. Soil P (Mehlich 1) varied from 1 to 6 ppm between 0 and 20 cm of soil depth, and from 1 to 4 ppm at the sub-superficial soil layer (20–40 cm). The minimum and maximum content of available P in the soil (Psoil) was 5.88 kg ha^− 1^ and 19.79 kg ha^− 1^.

Each experiment was arranged as a 12 × 10 alpha lattice design, with three complete replicates and ten incomplete blocks per replicate. Each block contained 12 plots, within which ten RILs (regular treatments) and the two parents (common checks) were allocated. Each plot consisted of a three-meter row, with 0.45 m between rows and 8 plants m^− 1^. Fertilization consisted of 150 kg ha^− 1^ of 20–00-20 (NPK) at sowing and 200 kg ha^− 1^ of urea applied 30 days after sowing.

Grain yield (Gy, kg ha^− 1^), flowering time (FT, days), plant height (PH, cm), phosphorus content in the plant (leaves and stems - Pp, kg ha^− 1^) and phosphorus content in the grain (Pg, kg ha^− 1^) were evaluated. For P measurements, samples of plant tissues and grains were collected in each plot, weighted and then dried at 65 °C to constant weight. Dry plant tissues and grains were then weighted, grounded and homogenized. Twenty gram - subsamples were used to determine P concentration and total P content (Pt), using inductively-coupled argon plasma emission spectrometry.

The phosphorus efficiency indexes were calculated according to the methodology proposed by Moll et al. [32], where: 1) phosphorus use efficiency (PUE) is equal to the product between phosphorus acquisition efficiency (PAE) and phosphorus internal utilization efficiency (PUTIL); 2) PAE is the total phosphorus content (Pt = Pp, P content in the plant + Pg, P content in the grain) divided by P content available in the soil; 3) PUTIL is Gy divided by Pt.

### Root system phenotyping in low-P conditions

Root morphology traits were assessed in nutrient solution as described by de Sousa et al. [49] and Hufnagel et al. [22], using a randomized block design with three replicates. Seeds were surface-sterilized using sodium hypochlorite (5%), washed with distilled water and placed in moistened paper rolls. After 4 days, uniform seedlings were transferred to moistened blotting papers and placed into paper pouches (24 × 33 × 0.02 cm) as described by Hund et al. [50].

Each experimental unit consisted of one pouch, with three plants per pouch, whose bottom (3 cm) was immersed in containers filled with 5 l of the nutrient solution described in [51], with pH 5.65 and a P concentration of 2.5 μM. The containers were kept in a growth chamber with 27 °C day and 20 °C night temperatures and a 12-h photoperiod, under continuous aeration for 13 days.

After 13 days, root images were acquired using a digital camera Nikon D300S SLR. Images were then analyzed using both the RootReader2D (http://www.plantmineralnutrition.net/software/rootreader2d/) and WinRhizo (http://www.regent.qc.ca/) software. The following traits were measured: root length (RL - cm); root diameter (RD - mm); total root surface area (SA - cm^2^); surface area of very fine roots between 0 and 1 mm in diameter (SA1 - cm^2^); surface area of fine roots between 1 and 2 mm in diameter (SA2 - cm^2^); surface area of thicker roots between 2 and 4.5 mm in diameter (SA3 - cm^2^); root volume (RV - cm^3^) and volume of fine roots between 1 and 2 mm in diameter (V2 - cm^3^). Shoot dry matter (SDM) and root dry matter (RDM), phosphorus content in the shoot (Ps) and phosphorus content in the root (Pr) (in grams) were also measured.

### Phenotypic analyses

Traits assessed in field and hydroponic experiments were analyzed using mixed models. For field experiments, the following model was used:

$$ {y}_{ijkl}=\mu +{E}_j+{R}_{k(j)}+{B}_{l(kj)}+{G}_i+{\varepsilon}_{ijkl} $$

*y*_*ijkl*_ is the phenotypic value of individual *i* in the block *l* of the *k*^*th*^ replicate, within the experiment *j*; *μ* is the overall mean; and *G*_*i*_ is the genetic effect of individual *i*, which can be defined as:

$$ {G}_i=\left\{\begin{array}{c}{g}_i\ i=1,\dots, {n}_g\ \\ {}{t}_ii={n}_g+1,\dots, {n}_g+{n}_c\end{array}\right. $$

*g*_*i*_ is the random effect of RIL *i*, *n*_*g*_ is the total number of RILs; *t*_*i*_ is the fixed effect of check *i*; and *n*_*c*_ is the total number of checks; *E*_*j*_ is the fixed effect of the *j*^*th*^ experiment (*j* = 1,  … , 4); *R*_*k*(*j*)_ is the fixed effect of replicate *k* (*k* = 1,  … , 3) in experiment *j*; *B*_*l*(*kj*)_ is the random effect of block *l* (*l* = 1,  … , 10) in the replicate *k*, within the experiment *j*; and ***ε*** = (*ε*_1111_, *ε*_2111_,  … , *ε*_*IJKL*_)^′^ is a *N*_*obs*_ × 1 residual random vector assumed to be normally distributed with mean zero and variance ${\sigma}_{\varepsilon}^2$, in which *N*_*obs*_ is the total number of observations.

The model used for analyzing the hydroponic experiments was:

$$ {y}_{ij}=\mu +{B}_j+{g}_i+{\varepsilon}_{ij} $$

where *y*_*ij*_ is the phenotypic value of the RIL *i* (*i* = 1,  … , *n*_*g*_) in the block *j*; *μ* is the overall mean; *g*_*i*_ is the random genetic effect of RIL *i*; *B*_*j*_ is the fixed effect of block *j* (*j* = 1,  … , 3); and ***ε*** = (*ε*_11_, *ε*_21_,  … , *ε*_*IJ*_)^′^ is a *N*_*obs*_ × 1 residual random vector assumed to be normally distributed with mean zero and variance ${\sigma}_{\varepsilon}^2$. Fixed and random effects were tested using the Wald statistics [52] and the likelihood ratio test (LRT, [53]) respectively, considering a 5% significance level (*α*).

For both statistical models, the genetic effect of RIL was first taken as random for estimating the genetic variance component (${\sigma}_g^2$) via restricted maximum likelihood (REML), and the heritability coefficient of each trait. The effect of RIL was then considered as fixed for estimating the adjusted means using best linear unbiased estimators (BLUEs). All the mixed models analyses were performed using the GenStat software (v.17.1.0) [54].

Trait heritabilities were estimated as proposed by Cullis et al. [55], called generalized heritabilities, using:

$$ {h}^2=1-\frac{\overline{v} BLUP}{2{\sigma}_g^2} $$

where $\overline{v} BLUP$ is the average variance of the difference between two best linear unbiased predictions (BLUPs). Person’s correlation coefficients [56] were estimated based on the adjusted means of genotypes for traits assessed in the field and in the hydroponic experiments, using the package *Hmic* [57] in R [58].

### SNP markers

Genomic DNA was isolated from approximately 500 mg of leaf tissue (eight plants per accession, i.e. RILs and their parents) as described by Saghai-Maroof et al. [59]. DNA samples were genotyped by sequencing according to Elshire et al. [60]. Reads were aligned to the version 1.4 of sorghum reference genome using Burrows-Wheeler Aligner program (BWA - [61]), and the SNP calling was performed using the GBS pipeline [62] implemented in the TASSEL software [63]. Missing genotypes were imputed using the NPUTE software [64]. Then, SNP data were filtered for 40% of minor allele frequency (MAF).

### QTL mapping

The final set of traits used for multi-trait QTL mapping was comprised of grain yield (Gy), surface area of fine roots in the 1–2 mm diameter class (SA2) and root diameter (RD). Multi-trait QTL mapping analysis was performed according to the procedures described in Silva et al. [65] implemented in R. For that, a multi-locus QTL mapping procedure was considered, using the Haley & Knott regression [66] and the following linear model:

$$ {y}_{ti}={\mu}_t+\sum \limits_{r=1}^m{a}_{tr}{x}_{ir}+{\varepsilon}_{ti} $$

where ***y***_*ti*_ is the adjusted mean of RIL *i* (*i* = 1,  … , *n*_*g*_) for trait *t* (*t* = 1,  … , *T*); *μ*_*t*_ is the intercept for each trait; *a*_*tr*_ is the *r*^*th*^ QTL main effect on trait *t*; *x*_*ir*_ represents the genotype of RIL *i* for the SNP marker *r* (*r* = 1, … , *n*_*M*_), being *n*_*M*_ the total number of markers; *x*_*ir*_ assumed values equal to 0 or 2 for RILs with homozygous genotypes for the allele donated by BR007B or SC283, respectively; and ***ε***_*i*_ = (*ε*_1*i*_, *ε*_2*i*_,  … , *ε*_*Ti*_)^′^ is a *T* × 1 random vector assumed to be independent and identically distributed according to a multivariate normal distribution with mean vector zero and positive definite symmetric variance-covariance matrix Σ_*ε*_, i.e. ***ε***_*i*_~*MVN*(0, Σ_*ε*_). Single-trait QTL mapping analyses were performed for each trait, using the above model (t = 1 gives an univariate regression model).

Multiple QTL models were built based on a forward-selection procedure, testing the significance of a putative QTL main effect at each SNP position along the genome. Significance of QTLs main effects were tested using the Score Statistic [67], considering a 10% significance level (*α*). According to simulations performed by Silva et al. [65], this significance level maximized the QTL detection power and kept the false discovery rate (i.e. the proportion of spurious QTLs) within an acceptable level.

QTL positions were refined after the inclusion of every new QTL in the model, until no more significant QTL main effects were found. Finally, non-significant QTL effects were removed from the model in a backward-elimination procedure, such as proposed in the seemingly unrelated regression coefficients method [68], considering a 1% significance level.

### Quantitative analysis of *SbPSTOL1* gene expression

Sorghum seedlings were grown in a modified Magnavaca nutrient solution [51] containing a low-P concentration (2.5 μM P), as described in the section *Root system phenotyping in low-P conditions***.** The experiment was set up in randomized block design with three replicates and three seedlings per experimental unit (paper pouch), giving a total of nine biological replicates per genotype. After 13 days in nutrient solution, the expression profiles of the *SbPSTOL1*-like genes (*Sb03g006765*, *Sb03g031690*, and *Sb07g002840*) were assessed in the roots of the RIL parents, BR007 and SC283. Total RNA was isolated from bulked root tissues (nine roots per bulk), using the SV Total RNA Isolation System kit (Promega Corporation, Madison, WI, USA), according to the manufacturer’s instructions. Total RNA (1 μg) was used for cDNA synthesis using the High Capacity cDNA Reverse Transcription kit (Applied Biosystems, Foster City, CA, USA). Transcripts were quantified by quantitative real-time PCR (qPCR-RT), using SYBR Green technology with the ABI Prism 7500 Fast System (Applied Biosystems, Foster City, CA, USA).

Transcript relative quantification was performed with 20 ng cDNA samples and 0.02 ng for the endogenous constitutive control (18 s rRNA). Primers were designed for *SbPSTOL1* and *18 s rRNA* sorghum genes using the PrimerQuest tool (https://www.idtdna.com/PrimerQuest/) (Additional file 7). Calculation of relative gene expressions were performed using the 2^-ΔΔCT^ method [69], with three technical replicates.

## Additional files

Additional file 1: Descriptive statistics and variance components for traits assessed in low-P conditions. (field and hydroponics). (DOCX 27 kb)

Additional file 2: Correlations and *p*-values among all traits assessed in low-P conditions. (field and hydroponics). (DOCX 30 kb)

Additional file 3: Single-trait QTL mapping profiles for grain yield (Gy), phosphorus use efficiency (PUE), phosphorus acquisition efficiency (PAE) and phosphorus internal utilization efficiency (PUTIL). Blue, light blue, pink and yellow inverted triangles depict the positions of QTLs for Gy, PUE, PAE and PUTIL respectively. (TIF 127 kb)

Additional file 4: Detailed information and confidence interval of the QTLs detected by single-trait QTL mapping. (Sheets “Single-trait values” and “Confidence Interval”. (XLSX 33 kb)

Additional file 5: Detailed information of the QTLs detected by multi-trait QTL mapping. (DOCX 24 kb)

Additional file 6: Synteny between sorghum, Arabidopsis and maize for the main QTLs detected in this study. (XLSX 55 kb)

Additional file 7: Primers used for qPCR-RT assays. (DOCX 14 kb)

## Abbreviations

Al: Aluminum
*ALMT1*: Aluminum-activated malate transporter in wheat
*ALS3*: Aluminum sensitive 3 (ABC-like transporter)
*Alt*_*SB*_: Aluminum tolerance locus in *Sorghum bicolor*
*AtSTOP1*: Sensitive to proton rhizotoxicity in Arabidopsis
BLUE: Best linear unbiased estimation
BLUP: Best linear unbiased predictions
BWA: Burrows-wheeler aligner program
DM: Dry matter
E2: Ubiquitin-conjugating enzyme
FT: Flowering time
GBS: Genotyping by sequencing
Gy: Grain yield
LRT: Likelihood rate test
MAF: Minimum allele frequency
Mb: Mega base pairs
*OsPSTOL1*: Phosphorus starvation tolerance 1 in rice
P: Phosphorus
PAE: Phosphorus use efficiency
Pg: Phosphorus content in grain
PH: Plant height
PHO2: Phosphate 2
Pp: Phosphorus content in the plant (leaves and stem)
Pr: Phosphorus content in root tissues
Ps: Phosphorus content in the shoot
Psoil: Phosphorus content available in the soil
Pt: Total phosphorus content
PUE: Phosphorus use efficiency
PUTIL: Phosphorus internal utilization efficiency
QTL: Quantitative trait locus
r: Correlation coefficient
RD: Root diameter
RDM: Root dry matter
REML: Restricted maximum likelihood
RIL: Recombinant inbreed line
RL: Root length
RV: Root volume
SA: Total root surface area
SA1: Surface area of very fine roots between 0 and 1 mm in diameter
SA2: Surface area of fine roots between 1 and 2 mm in diameter
SA3: Surface area of thicker roots between 2 and 4.5 mm in diameter
*SbMATE*: Multidrug and toxic compound extrusion in *Sorghum bicolor*
*SbPSTOL1*: Phosphorus starvation tolerance1 in *Sorghum bicolor*
SDM: Shoot dry matter
SNP: Single nucleotide polymorphism
SRL: Specific root length
UBC24: Ubiquitin-conjugating 24 enzyme
V2: Volume of fine roots between 1 and 2 mm in diameter
