# Supplementary material for: The genetic architecture of phosphorus efficiency in sorghum involves pleiotropic QTL for root morphology and grain yield under low phosphorus availability in the soil
Source: BMC Plant Biol. 2019 Feb 28;19:87. doi: 10.1186/s12870-019-1689-y (PMC6394046; doi:10.1186/s12870-019-1689-y)
Supplement: Supplementary file 1 — Descriptive statistics and variance components for traits assessed in low-P conditions. (field and hydroponics). (DOCX 27 kb) [file 12870_2019_1689_MOESM1_ESM.docx]

**Additional file 1** Descriptive statistics and variance components for traits assessed in low P conditions (field and hydroponics)

| **Variance components** | **Field traits** | | | | |  | **Traits assessed in hydroponics** | | | | | | | | | | | | |
| --- | --- | --- | --- | --- | --- | --- | --- | --- | --- | --- | --- | --- | --- | --- | --- | --- | --- | --- | --- |
|  |  |  |  |  |  |  | **Root morphology** | | | | | | | |  | **Dry matter / P content** | | | |
|  | **Gy** | **FT** | **PH** | **Pp** | **Pg** |  | **RL** | **RD** | **SA** | **SA1** | **SA2** | **SA3** | **RV** | **V2** |  | **SDM** | **RDM** | **Ps** | **Pr** |
|  | **(kg ha^-1^)** | **(days)** | **(cm)** | **(kg ha^-1^)** | **(kg ha^-1^)** |  | **(cm)** | **(mm)** | **(cm²)** | **(cm²)** | **(cm²)** | **(cm²)** | **(cm³)** | **(cm3)** |  | **(g)** | **(g)** | **(g)** | **(g)** |
| Genetic variance | 187809 | 3.62 | 222.20 | 0.09 | 0.59 |  | 2350.00 | 5.36×10^-4^ | 71.47 | 43.87 | 1.16 | 0.01 | 0.01 | 1.15×10^-3^ |  | 1.33×10^-5^ | 7.73×10^-6^ | 1.42×10^-4^ | 1.52×10^-4^ |
| Residual variance | 467203 | 3.98 | 149.40 | 0.44 | 1.90 |  | 1963.00 | 3.08×10^-3^ | 67.33 | 42.41 | 2.60 | 0.02 | 0.02 | 2.47×10^-3^ |  | 1.11×10^-5^ | 2.64×10^-5^ | 3.40×10^-4^ | 1.60×10^-4^ |
| **RIL population** | | | | | | | | | | | | | | | | | | | |
| Mean | 2081 | 70.41 | 141.10 | 1.26 | 4.24 |  | 190.00 | 0.63 | 36.85 | 28.71 | 4.23 | 0.29 | 0.58 | 0.13 |  | 0.02 | 0.02 | 0.06 | 0.05 |
| Minimum | 132 | 66.63 | 75.20 | 0.24 | 0.53 |  | 54.50 | 0.46 | 11.47 | 8.46 | 1.61 | 0.02 | 0.19 | 0.05 |  | 0.01 | 0.01 | 0.02 | 0.02 |
| Maximum | 4562 | 77.64 | 222.00 | 3.37 | 7.96 |  | 337.30 | 0.74 | 65.51 | 50.16 | 9.84 | 0.63 | 1.03 | 0.31 |  | 0.03 | 0.03 | 0.12 | 0.10 |
| h² | 0.54 | 0.74 | 0.83 | 0.38 | 0.48 |  | 0.77 | 0.34 | 0.75 | 0.75 | 0.56 | 0.53 | 0.65 | 0.57 |  | 0.78 | 0.46 | 0.55 | 0.73 |
| **Parents** | | | | | | | | | | | | | | | | | | | |
| BR007 | 1965 | 71.35 | 147.00 | 1.14 | 3.78 |  | 233.50 | 0.59 | 42.52 | 33.99 | 3.81 | 0.21 | 0.63 | 0.11 |  | 0.02 | 0.02 | 0.07 | 0.04 |
| SC283 | 2196 | 69.47 | 135.20 | 1.38 | 4.70 |  | 176.60 | 0.68 | 37.18 | 27.15 | 6.17 | 0.30 | 0.63 | 0.19 |  | 0.02 | 0.02 | 0.07 | 0.05 |

Gy: grain yield; FT: flowering time; PH: plant height; Pp: phosphorus content in the plant (leaves and stem); Pg: phosphorus content in the grain; RL: root length; RD: root diameter; SA: total root surface area; SA1: surface area of very fine roots between 0-1 mm in diameter, SA2: surface area of fine roots between 1-2 mm in diameter; SA3: surface area of thicker roots between 2-4.5 mm in diameter; RV: root volume; V2 volume of fine roots between 1-2 mm in diameter; SDM: shoot dry matter; RDM: root dry matter; Ps: phosphorus content in the shoot; Pr: phosphorus content in the root. All genetic variance components were significant at p-values $\leq$ 0.05 by the Likelihood Ratio Test. h²: heritability. Best linear unbiased predictors (BLUEs) are shown for the parents.
