# Supplementary material for: The genetic architecture of phosphorus efficiency in sorghum involves pleiotropic QTL for root morphology and grain yield under low phosphorus availability in the soil
Source: BMC Plant Biol. 2019 Feb 28;19:87. doi: 10.1186/s12870-019-1689-y (PMC6394046; doi:10.1186/s12870-019-1689-y)
Supplement: Supplementary file 2 — Correlations and p-values among all traits assessed in low-P conditions. (field and hydroponics). (DOCX 30 kb) [file 12870_2019_1689_MOESM2_ESM.docx]

**Additional file** **2** Correlations and p-values among all traits assessed in low P conditions (field and hydroponics)

|  | **p-values** | | **Field traits** | | | | | | | | | **Traits assessed in hydroponics** | | | | | | | | | | | | | |
| --- | --- | --- | --- | --- | --- | --- | --- | --- | --- | --- | --- | --- | --- | --- | --- | --- | --- | --- | --- | --- | --- | --- | --- | --- | --- |
|  |  |  |  |  |  |  |  |  |  |  |  | **Root morphology** | | | | | | | | | **Dry matter / P content** | | | | |
| **Correlations** | |  | **Gy** | **HI** | **FT** | **PH** | **Pp** | **Pg** | **PAE** | **PUTIL** | **PUE** | **RL** | **SA** | **RD** | **RV** | **V2** | **SA1** | **SA2** | **SA3** | **SDM** | | **RDM** | **RSR** | **Ps** | **Pr** |
| **Field traits** | | **Gy** |  | 0.00 | 0.00 | 0.00 | 0.00 | 0.00 | 0.00 | 0.00 | 0.00 | 0.04 | 0.03 | 0.19 | 0.03 | 0.04 | 0.05 | 0.04 | 0.10 | 0.02 | | 0.01 | 0.98 | 0.01 | 0.03 |
|  |  | **HI** | 0.42 |  | 0.69 | 0.00 | 0.00 | 0.00 | 0.02 | 0.00 | 0.00 | 0.00 | 0.00 | 0.00 | 0.00 | 0.00 | 0.00 | 0.00 | 0.00 | 0.45 | | 0.07 | 0.55 | 0.07 | 0.04 |
|  |  | **FT** | -0.14 | -0.02 |  | 0.00 | 0.06 | 0.00 | 0.00 | 0.52 | 0.01 | 0.04 | 0.07 | 0.02 | 0.17 | 0.13 | 0.02 | 0.18 | 0.13 | 0.42 | | 0.10 | 0.18 | 0.04 | 0.03 |
|  |  | **PH** | 0.43 | 0.25 | -0.28 |  | 0.24 | 0.00 | 0.00 | 0.00 | 0.00 | 0.15 | 0.31 | 0.08 | 0.63 | 0.28 | 0.19 | 0.35 | 0.51 | 0.01 | | 0.00 | 0.99 | 0.04 | 0.00 |
|  |  | **Pp** | 0.23 | -0.52 | -0.10 | 0.06 |  | 0.00 | 0.00 | 0.00 | 0.00 | 0.00 | 0.00 | 0.00 | 0.00 | 0.00 | 0.00 | 0.00 | 0.00 | 0.03 | | 0.00 | 0.23 | 0.01 | 0.55 |
|  |  | **Pg** | 0.92 | 0.38 | -0.14 | 0.40 | 0.21 |  | 0.00 | 0.00 | 0.00 | 0.07 | 0.06 | 0.28 | 0.06 | 0.06 | 0.08 | 0.05 | 0.07 | 0.00 | | 0.00 | 0.48 | 0.00 | 0.00 |
|  |  | **PAE** | 0.85 | 0.11 | -0.15 | 0.34 | 0.56 | 0.90 |  | 0.20 | 0.00 | 0.00 | 0.00 | 0.01 | 0.00 | 0.01 | 0.01 | 0.01 | 0.01 | 0.01 | | 0.00 | 0.80 | 0.00 | 0.01 |
|  |  | **PUTIL** | 0.40 | 0.63 | -0.03 | 0.22 | -0.52 | 0.17 | -0.06 |  | 0.00 | 0.02 | 0.02 | 0.04 | 0.05 | 0.14 | 0.02 | 0.15 | 0.01 | 0.13 | | 0.07 | 0.94 | 0.14 | 0.15 |
|  |  | **PUE** | 0.97 | 0.40 | -0.14 | 0.37 | 0.25 | 0.89 | 0.87 | 0.38 |  | 0.06 | 0.05 | 0.14 | 0.05 | 0.07 | 0.07 | 0.06 | 0.16 | 0.05 | | 0.03 | 0.92 | 0.01 | 0.27 |
| **Traits assessed in hydroponics** | **Root morphology** | **RL** | 0.10 | -0.22 | -0.10 | 0.07 | 0.23 | 0.09 | 0.15 | -0.12 | 0.10 |  | 0.00 | 0.00 | 0.00 | 0.00 | 0.00 | 0.00 | 0.00 | 0.00 | | 0.00 | 0.34 | 0.00 | 0.01 |
|  |  | **SA** | 0.11 | -0.21 | -0.09 | 0.05 | 0.22 | 0.10 | 0.15 | -0.11 | 0.10 | 0.98 |  | 0.00 | 0.00 | 0.00 | 0.00 | 0.00 | 0.00 | 0.00 | | 0.00 | 0.13 | 0.00 | 0.00 |
|  |  | **RD** | -0.07 | 0.17 | 0.12 | -0.09 | -0.20 | -0.05 | -0.13 | 0.10 | -0.07 | -0.61 | -0.46 |  | 0.00 | 0.09 | 0.00 | 0.05 | 0.00 | 0.20 | | 0.00 | 0.02 | 0.00 | 0.00 |
|  |  | **RV** | 0.11 | -0.18 | -0.07 | 0.02 | 0.20 | 0.10 | 0.14 | -0.10 | 0.10 | 0.92 | 0.98 | -0.28 |  | 0.00 | 0.00 | 0.00 | 0.00 | 0.00 | | 0.00 | 0.04 | 0.00 | 0.00 |
|  |  | **V2** | 0.10 | -0.15 | 0.08 | -0.05 | 0.17 | 0.10 | 0.13 | -0.07 | 0.09 | 0.61 | 0.72 | 0.09 | 0.82 |  | 0.00 | 0.00 | 0.00 | 0.00 | | 0.00 | 0.01 | 0.00 | 0.00 |
|  |  | **SA1** | 0.10 | -0.21 | -0.11 | 0.07 | 0.21 | 0.09 | 0.14 | -0.11 | 0.09 | 0.99 | 0.99 | -0.53 | 0.95 | 0.62 |  | 0.00 | 0.00 | 0.00 | | 0.00 | 0.28 | 0.00 | 0.00 |
|  |  | **SA2** | 0.11 | -0.15 | 0.07 | -0.05 | 0.16 | 0.10 | 0.13 | -0.07 | 0.10 | 0.61 | 0.72 | 0.10 | 0.82 | 1.00 | 0.63 |  | 0.00 | 0.00 | | 0.00 | 0.01 | 0.00 | 0.00 |
|  |  | **SA3** | 0.08 | -0.18 | -0.08 | 0.03 | 0.22 | 0.09 | 0.14 | -0.13 | 0.07 | 0.58 | 0.60 | -0.23 | 0.60 | 0.50 | 0.57 | 0.49 |  | 0.00 | | 0.00 | 0.30 | 0.00 | 0.00 |
|  | **Dry matter / P content** | **SDM** | 0.12 | -0.04 | -0.04 | 0.13 | 0.11 | 0.15 | 0.14 | -0.08 | 0.10 | 0.62 | 0.68 | -0.06 | 0.72 | 0.64 | 0.64 | 0.65 | 0.55 |  | | 0.00 | 0.00 | 0.00 | 0.00 |
|  |  | **RDM** | 0.14 | -0.09 | -0.08 | 0.15 | 0.17 | 0.15 | 0.17 | -0.09 | 0.11 | 0.72 | 0.77 | -0.20 | 0.78 | 0.66 | 0.74 | 0.67 | 0.62 | 0.78 | |  | 0.01 | 0.00 | 0.00 |
|  |  | **RSR** | 0.00 | -0.03 | -0.07 | 0.00 | 0.06 | -0.04 | 0.01 | 0.00 | 0.00 | -0.05 | -0.08 | -0.12 | -0.10 | -0.14 | -0.05 | -0.14 | -0.05 | -0.49 | | 0.125 |  | 0.00 | 0.17 |
|  |  | **Ps** | 0.14 | -0.09 | -0.10 | 0.10 | 0.14 | 0.15 | 0.16 | -0.07 | 0.12 | 0.61 | 0.63 | -0.18 | 0.64 | 0.56 | 0.60 | 0.56 | 0.47 | 0.74 | | 0.64 | -0.29 |  | 0.00 |
|  |  | **Pr** | 0.11 | 0.10 | -0.11 | 0.17 | 0.03 | 0.17 | 0.13 | -0.07 | 0.06 | 0.12 | 0.18 | 0.16 | 0.24 | 0.29 | 0.15 | 0.30 | 0.21 | 0.43 | | 0.52 | 0.07 | 0.40 |  |

Gy: grain yield; HI: harvest index; FT: flowering time; PH: plant height; Pp: phosphorus content in the plant (leaves and stem); Pg: phosphorus content in the grain; PAE: phosphorus acquisition efficiency; PUTIL: phosphorus internal utilization efficiency; PUE: phosphorus use efficiency; RL: root length; SA: total root surface area; RD: root diameter; RV: root volume; V2 volume of fine roots between 1-2 mm in diameter; SA1: surface area of very fine roots between 0-1 mm in diameter. SA2: surface area of fine roots between 1-2 mm in diameter; SA3: surface area of thicker roots between 2-4.5 mm in diameter; SDM: shoot dry matter; RDM: root dry matter; RSR: root shoot ratio; Ps: phosphorus content in the shoot; Pr: phosphorus content in the root.
