# Supplementary figures and images for: The genetic architecture of phosphorus efficiency in sorghum involves pleiotropic QTL for root morphology and grain yield under low phosphorus availability in the soil
Source: BMC Plant Biol. 2019 Feb 28;19:87. doi: 10.1186/s12870-019-1689-y (PMC6394046; doi:10.1186/s12870-019-1689-y)

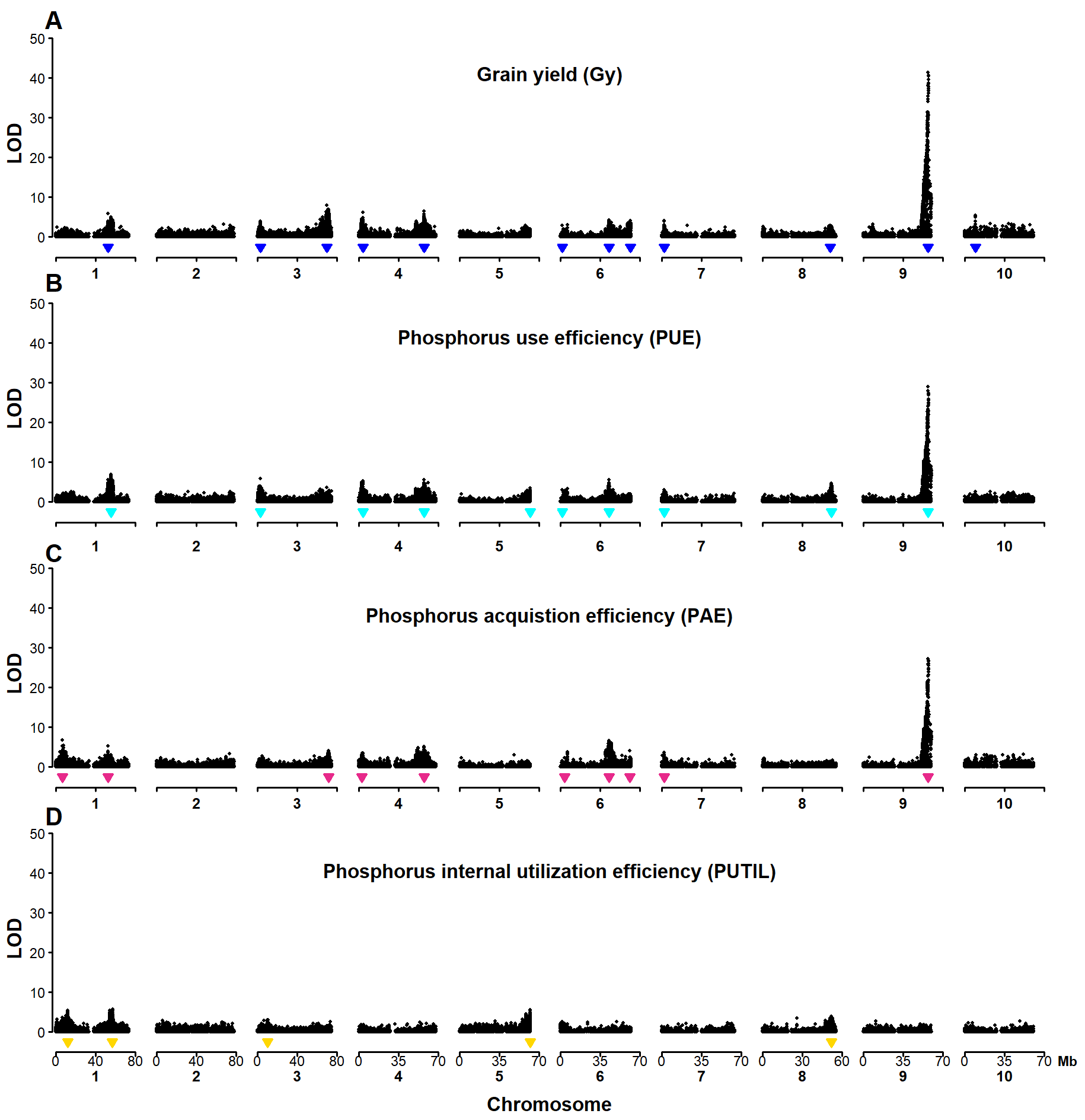

Supplement: Supplementary file 3 — Single-trait QTL mapping profiles for grain yield (Gy), phosphorus use efficiency (PUE), phosphorus acquisition efficiency (PAE) and phosphorus internal utilization efficiency (PUTIL). Blue, light blue, pink and yellow inverted triangles depict the positions of QTLs for Gy, PUE, PAE and PUTIL respectively. (TIF 127 kb) [file 12870_2019_1689_MOESM3_ESM.tif]
