# Supplementary material for: The genetic architecture of phosphorus efficiency in sorghum involves pleiotropic QTL for root morphology and grain yield under low phosphorus availability in the soil
Source: BMC Plant Biol. 2019 Feb 28;19:87. doi: 10.1186/s12870-019-1689-y (PMC6394046; doi:10.1186/s12870-019-1689-y)
Supplement: Supplementary file 5 — Detailed information of the QTLs detected by multi-trait QTL mapping. (DOCX 24 kb) [file 12870_2019_1689_MOESM5_ESM.docx]

**Additional file 5** Detailed information of the QTLs detected by multi-trait QTL mapping

| **QTL** | **Position (bp)** | **Inferior limit (SNP - bp)^a^** | **Upper limit (SNP - bp)^b^** | **Criterion^c^** | **Effect^d^** | | | **h² _QTL_ (%)^e^** | | | **LOD^f^** |
| --- | --- | --- | --- | --- | --- | --- | --- | --- | --- | --- | --- |
|  |  |  |  |  | **Gy** | **RD** | **SA2** | **Gy** | **RD** | **SA2** |  |
| *Gy-1* | 55802244 | 55133968 | 56732121 | LOD-3 | 0.22 | - | - | 4.76 | - | - | 8.18 |
| *RD/SA2-2* | 65506207 | 63980832 | 67203635 | LOD-5 | - | 0.20 | 0.29 | - | 3.96 | 8.25 | 11.56 |
| *Gy-3* | 1617914 | 1240416 | 202842 | LOD-1.5 | 0.13 | - | - | 1.75 | - | - | 3.19 |
| *SA2-3* | 745866 | 6872695 | 7960595 | LOD-5 | - | - | 0.27 | - | - | 7.19 | 7.52 |
| *Gy/SA2-3* | 71015638 | 70804761 | 71852763 | LOD-5 | 0.21 | - | 0.23 | 4.26 | - | 5.30 | 10.34 |
| *Gy-4.1* | 4059198 | 4059197 | 4060107 | Adjacent SNP | -0.13 | - | - | 1.83 | - | - | 3.29 |
| *Gy-4.2* | 57829755 | 56152948 | 60859112 | LOD-5 | 0.18 | - | - | 3.03 | - | - | 5.13 |
| *RD-5* | 5541663 | 55259386 | 55655665 | LOD-1.5 | - | 0.20 | - | - | 3.80 | - | 3.44 |
| *Gy-6.1* | 1515389 | 1471969 | 1574949 | LOD-3 | -0.15 | - | - | 2.17 | - | - | 3.38 |
| *Gy-6.2* | 42531422 | 42316732 | 44569495 | LOD-3 | -0.19 | - | - | 3.58 | - | - | 5.46 |
| *Gy-6.3* | 61839737 | 61183372 | 61968898 | LOD-3 | -0.18 | - | - | 3.24 | - | - | 5.59 |
| *Gy/RD-7* | 3672580 | 2132016 | 4813345 | LOD-5 | 0.14 | 0.15 | - | 2.01 | 2.36 | - | 5.90 |
| *SA2-8* | 1093993 | 891531 | 1206164 | LOD-1.5 | - | - | -0.20 | - | - | 3.97 | 4.31 |
| *Gy-9* | 57236934 | 56918847 | 57337475 | LOD-3 | -0.50 | - | - | 24.73 | - | - | 35.66 |

^a^Inferior limit for confidence interval

^b^Upper limit for confidence interval

^c^Criterion adopted to determine of confidence interval

^d^Positive effect indicates that favorable allele was donated by SC283, and negative effect indicates favorable allele donated by BR007. Trait values were standardized by subtracting from each value the trait mean and dividing by the trait standard deviation.

^e^The proportion of the genetic variance explained by each QTL (h² QTL, %).

^f^Log-of-the odds

Chr: chromosome; Gy: grain yield (kg ha^-1^); RD: root diameter (mm); SA2: surface area of fine roots between 1-2 mm in diameter (cm²). The first number after of the trace (-) in the codification of the QTLs indicates the chromosome where they were mapped and the second number distinguish QTLs mapped in the same chromosome.
