# Supplementary material for: The genetic architecture of phosphorus efficiency in sorghum involves pleiotropic QTL for root morphology and grain yield under low phosphorus availability in the soil
Source: BMC Plant Biol. 2019 Feb 28;19:87. doi: 10.1186/s12870-019-1689-y (PMC6394046; doi:10.1186/s12870-019-1689-y)
Supplement: Supplementary file 7 — Primers used for qPCR-RT assays. (DOCX 14 kb) [file 12870_2019_1689_MOESM7_ESM.docx]

**Additional file 7** Primers used for qPCR-RT assays

| **Gene ID** | **Primer ID** | **Sequence 5'- 3'** |
| --- | --- | --- |
| *Sb03g006765* | RTPupSbH602  RTPupSbH701 | CGCCGACGATGAACATCTC  TTGGCTCTGCTGAAGACGAA |
| *Sb03g031690* | RTPupSb504  RTPupSb601 | CGCTCCTCCTTGCTGTCTTG  TGTAATCGTCGTCGGAAGGAT |
| *Sb07g002840* | RTPupSbH102  RTPupSbH103 | CACCAGCCTCGATTTCATACAA  AGCCGCACCGGAAGTAGAC |
| *18s rRNA* | Sb18s_F  Sb18s_R | AATCCCTTAACGAGGATCCATTG  CGCTATTGGAGCTGGAATTACC |
